# A suite of automated tools to quantify hand and wrist motor function after cervical spinal cord injury

**DOI:** 10.1186/s12984-019-0518-8

**Published:** 2019-04-11

**Authors:** Katelyn M. Grasse, Seth A. Hays, Kimiya C. Rahebi, Victoria S. Warren, Elizabeth A. Garcia, Jane G. Wigginton, Michael P. Kilgard, Robert L. Rennaker

**Affiliations:** 1 The University of Texas at Dallas, Texas Biomedical Device Center800 West Campbell Road, Richardson, TX 75080-3021 USA; 2 The University of Texas at Dallas, Erik Jonsson School of Engineering and Computer Science, 800 West Campbell Road, Richardson, TX 75080-3021 USA; 3 The University of Texas at Dallas, School of Behavioral Brain Sciences, 800 West Campbell Road, Richardson, TX 75080-3021 USA

**Keywords:** Spinal cord injury, Prehension, Force, Range of motion, Hand, Wrist, Assessment

## Abstract

**Background:**

Cervical spinal cord injury (cSCI) often causes chronic upper extremity disability. Reliable measurement of arm function is critical for development of therapies to improve recovery after cSCI. In this study, we report a suite of automated rehabilitative tools to allow simple, quantitative assessment of hand and wrist motor function.

**Methods:**

We measured range of motion and force production using these devices in cSCI participants with a range of upper limb disability and in neurologically intact participants at two time points separated by approximately 4 months. Additionally, we determined whether measures collected with the rehabilitative tools correlated with standard upper limb assessments, including the Graded Redefined Assessment of Strength, Sensibility, and Prehension (GRASSP) and the Jebsen Hand Function Test (JHFT).

**Results:**

We find that the rehabilitative devices are useful to provide assessment of upper limb function in physical units over time in SCI participants and are well-correlated with standard assessments.

**Conclusions:**

These results indicate that these tools represent a reliable system for longitudinal evaluation of upper extremity function after cSCI and may provide a framework to assess the efficacy of strategies aimed at improving recovery of upper limb function.

**Electronic supplementary material:**

The online version of this article (10.1186/s12984-019-0518-8) contains supplementary material, which is available to authorized users.

## Introduction

Spinal cord injury is a common cause of disability, affecting more than 300,000 people in the US [1]. The majority of injuries occur at the cervical level, which often impairs function of upper extremities and can lead to chronic disability [2–4]. Accurate and sensitive measurement of upper limb function is a critical part of the development and assessment of new therapies to improve recovery after SCI.

The most common method of arm assessment after cSCI involves subjective ordinal scoring of motor function by a skilled examiner [5–7]. While this approach yields rapid and reliable results, ordinal assessment may not always be sensitive to small improvements that can be functionally meaningful [8, 9]. Objective measurement may provide improved measurement sensitivity, and as sensor technologies have become smaller and more accessible, numerous tools have been developed for assessing arm function after cSCI [10]. Dynamometers and myometers are widely used to measure isometric force of isolated arm functions in continuous physical units [11, 12]. Other assessment tools measure position and force during execution of simulated functional tasks [13, 14]. These systems indeed provide greater precision or a more direct characterization of functional ability than traditional measures, but the continued reliance upon ordinal assessment indicates an unmet clinical need for the development of measurement technologies.

To increase their utility and implementation, measurement systems should be simple to use, facilitate standardized administration, report sensitive, quantitative metrics, and provide reliable longitudinal testing [15]. We designed a suite of modular rehabilitative devices for objective assessment of various isolated hand and wrist motor functions to address these needs. In addition to the measurement modules, a table-mounted armrest with simple, interchangeable tasks facilitates standardized administration across a wide range of arm impairments [11]. The system records measurements in continuous physical units, providing unambiguous results across many aspects of function. Furthermore, the devices enable collection of multiple trials to increase sensitivity and reliability of measurements.

In this study, we tested whether these tools could quantify motor impairments in individuals with cSCI, determined measurement detection limits, and examined retest reliability by assessing the same participants 4 months later. We also established concurrent validity by correlating performance on the rehabilitative devices with two common metrics of upper limb function after cSCI, the Graded Refined Assessment of Strength, Sensibility, and Prehension (GRASSP) exam and the Jebsen Hand Function Test (JHFT) [5]. Our results demonstrate that the system provides reliable measurements over time and that performance correlates with established outcome measures. The results indicate that the novel system can deliver simple and reliable longitudinal evaluation of upper extremity function after cSCI and may provide a framework to assess the efficacy of strategies aimed at improving recovery of upper limb function.

## Methods

### Study design

All procedures were approved by the Institutional Review Board at the University of Texas at Dallas. Fifteen participants with cSCI and no known cognitive deficits were recruited, and nine completed all components of the study. All participants had motor impairments in the right upper limb with some residual function. Participants with cSCI were assessed on at least four separate occasions, with each session lasting between 30 min and 2 h according to individual needs. In the first session, participants were informed about the study plan and gave consent (*n* = 15). In the second session, the participant’s right arm was assessed with the rehabilitative devices (*n* = 13). The participant then completed the GRASSP (*n* = 11) and Jebsen Hand Function (*n* = 12) assessments for both hands. In the third session, occurring approximately 4 months later, right arm function was retested with the rehabilitative devices (*n* = 10). In the fourth session, a complete ASIA exam and any remaining assessments were administered by a licensed physical therapist (*n* = 9) [5, 6, 16].

Thirteen participants with no history of neurological or arm injury were recruited through the University of Texas at Dallas. Within one half-hour session, these participants gave consent and completed assessments using the rehabilitative devices with their right arm only. This testing was repeated on the right arm approximately 4 months later. Arm assessments of all participants were video recorded.

### Device hardware and software

**Fig. 1:**
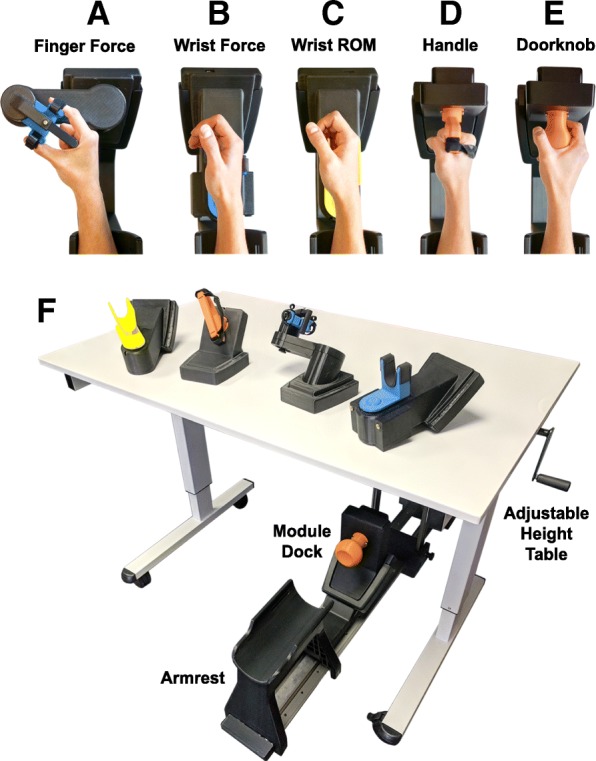
Suite of hand and wrist assessment devices. Images of the finger flexion/extension device (**a**), the wrist flexion/extension devices (**b/c**), the wrist rotation device with the D-grip handle (**d**) and with the doorknob manipulandum (**e**), and the testing table used for assessing cSCI participants (**f**)

The system consisted of seven devices that were each designed to measure either the force or range of motion (ROM) of simple hand and wrist movements (Fig. 1). System components were designed using the CAD program SolidWorks (Dassault Systèmes) and created with Dimension Elite and Fortus 250mc 3D printers (Stratasys). Four isometric tasks were designed to measure force and prevented joint movement. The isometric tasks assessed finger force, wrist flexion/extension force, and wrist rotation force with a D-grip handle and doorknob manipulandum. Three isotonic tasks were designed to assess ROM of a single joint with negligible resistance. The isotonic tasks measured wrist flexion/extension range of motion and wrist rotation range of motion with the handle and doorknob manipulanda.

**Fig. 2:**
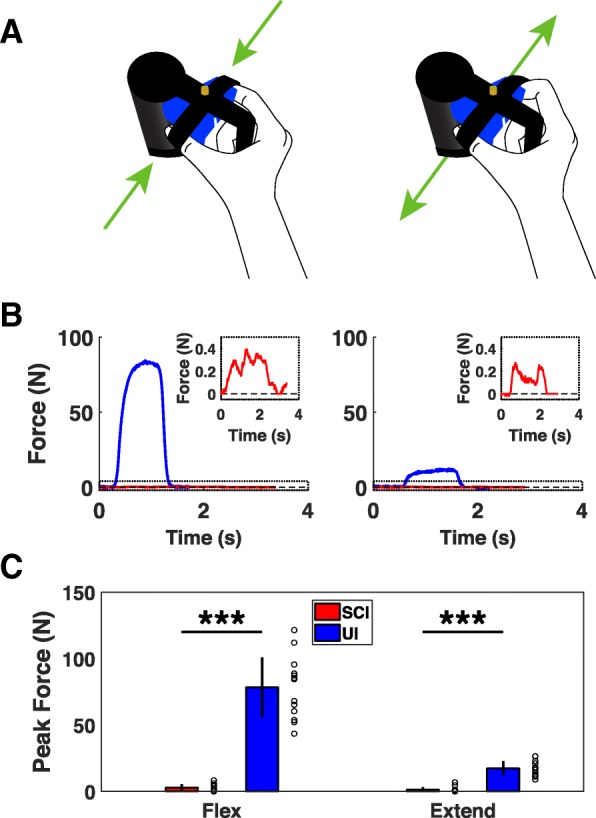
Device for assessing finger force. **a** Diagram of isometric finger module illustrating the force directions. **b** Example of single finger flexion and extension trials from uninjured and cSCI participants. Inset shows details lower force production in cSCI participant. **c** cSCI participants generate significantly lower finger flexion and extension forces compared to uninjured controls. Individual data is depicted with open circles. Error bars indicate SD. Significant differences were determined by Wilcoxon rank sum tests and are noted as ***p < 0.001

Mounted firmly within the 3D-printed housing of each device was either a quadrature rotary encoder for measuring angle in ROM devices (TRD-S360VD, AutomationDirect.com) or two 20 kg capacity load cells for measuring force in isometric devices (RB-Phi-119, RobotShop.com). The rotary encoders provided 0.25° resolution, and the load cells provided 0.02 N or 0.007 Nm resolution for the pinch and other force modules, respectively. The load cells in the finger force device were each calibrated with weights, while the other force devices were calibrated using a commercial torque meter (GLK-250E, Imada.com). Calibration constants were linearly interpolated and saved with a custom Matlab program. Each device contained a custom printed circuit board (PCB) to automate device identification by the software and process the signals from the rotary encoder or load cells. A second PCB and a microcontroller (Arduino Uno, Arduino.cc) sampled the rotary encoder or load cells at 125 Hz and sent timestamped signals to a custom Matlab assessment program that captured, saved, and displayed data.

Figure 1 depicts the position of the hand in each of the devices. For testing with the finger force device, Velcro straps were wrapped around the distal phalanges of the thumb and index fingers, securing the fingertips 5 cm apart (Fig. 1a). An adjustable plastic bar was extended to meet the area between the thumb and index finger to stabilize hand position. For the wrist force device, the hand was aligned so that torque was measured from the ulnar styloid process and to a point 5 cm distal, where the ulnar aspect of the hand rested in a stationary cradle (Fig. 1b) [17, 18]. For both devices utilizing the handle manipulandum, the palm was firmly affixed to the handle with Velcro straps that pressed against the dorsal and distal end of the metacarpals (Fig. 1d). For testing with the doorknob manipulandum, participants who could not produce thumb opposition were allowed to position the palm and fingers around the doorknob in a way that would provide sufficient grip stability.

### Testing with the rehabilitative devices

To administer testing, the rehabilitative devices were affixed in place to an adjustable-height table (Fig. 1f). Participants were oriented in front of the table, and the forearm was comfortably positioned and rested in the armrest to an elbow flexion angle of 90° and shoulder abduction angle less than 45° with shoulders level. Each assessment began with neutral wrist pronation and flexion angles of 0°.

All participants completed the seven tasks in the same order, starting with four isometric force tasks (wrist rotation with the doorknob, wrist flexion/extension, wrist rotation with the D-grip handle, then finger flexion/extension) and followed by three isotonic ROM tasks (wrist rotation with the doorknob, wrist flexion/extension, then wrist rotation with the D-grip handle). For each of these tasks, participants performed 10 trials in each direction of movement (flexion/extension or pronation/supination), starting with the inward anterior direction (i.e., flexion or pronation). For isometric force testing, participants performed five trials in each direction and then repeated this sequence after a minute of rest. For ROM testing, participants completed ten consecutive trials in each direction. Participants were instructed to give their best effort on each trial, then return to and relax at the neutral position between trials. Any trials with obvious compensatory movement were excluded and repeated after providing verbal feedback.

### Standard assessments

cSCI participants were tested with the GRASSP and JHFT exams, two established functional arm assessments suited to the injury [5]. Both assessments were administered by trained medical professionals with certified equipment and according to standard procedures [7, 19]. The total GRASSP score ranges from 0 to 116 points for one arm and is comprised of the sum of four subscores that quantify muscle strength, finger sensation, grip dexterity and functional task performance. The JHFT score is the total time taken to complete seven common activities of daily living using one arm, allowing up to 2 min per task for a maximum total score of 14 min. The GRASSP and JHFT scores for the right arm were compared to the measurements made with the devices. Commercial isometric strength gauges were used in the rehabilitative device test sessions to measure peak grip and pinch force for the right hand. A digital dynamometer (Fabrication Enterprises Inc., 1335 N capacity, 4.45 N resolution) was used to measure power grip strength and a digital pinch gauge (Fabrication Enterprises Inc., 222 N capacity, 0.445 N resolution) was used to measure lateral pinch force. Three trials were acquired and averaged with each device [20].

### Statistics

All data represent measurements from the right arm and are presented in the text as mean ± standard deviation (SD). For measurements made with the devices, maximum force or angle from each of the 10 trials on each task was averaged to produce an individual mean and SD for each participant. Individual means are displayed as open circles in the figures. Coefficient of variation (CV), the ratio of the SD of the 10 trials to the mean, was calculated for each individual on each task. Minimally detectable differences (MDD) and intraclass correlation coefficients (ICC) were calculated as described by Beckerman et al. [21]. For each metric, the MDD was compared to the average SD of all 20 trials acquired from both test sessions to determine the effect of trial count on measurement sensitivity. A composite score of performance on the devices was calculated to simplify correlations. To calculate the composite score, performance on each of the 14 metrics (2 movement directions from each of the 7 devices) was normalized to the mean value for the corresponding metric in uninjured subjects. The normalized means for each metric were then averaged, producing a score between 0 and 1 for each individual, with 0 representing no motor function and 1 representing function equivalent to the mean of control participants. A composite score was only calculated for participants that provided measurements on all metrics, leading to exclusion of one participant with cSCI who was not able to use the finger force device. To simplify correlations between the individual devices and standard assessments, the two movement directions for each device were added together. Significant differences were determined using unpaired t-tests, paired t-tests, Wilcoxon rank sum tests, and Pearson correlations, as appropriate. The statistical test used for each comparison is noted in the text. In all figures, * indicates *p* < 0.05, ** indicates *p* < 0.01, and *** indicates *p* < 0.001. Error bars indicate mean ± SD in all figures.

## Results

### Participants

Demographics for cSCI participants are provided in Table 1. Injury levels ranged from C4 to C7, with the majority confirmed as incomplete by an ASIA exam. Approximately 30% (*n* = 4) of both groups of participants were female. The average age, weight, and height was similar in cSCI and uninjured control (UI) participants (Age; cSCI: 32.2 ± 3.7, UI: 28.9 ± 0.9; Unpaired t-test, *p* = 0.41; Height; cSCI: 1.76 ± 0.04 m, UI: 1.74 ± 0.04 m; *p* = 0.73; Weight; cSCI: 75.9 ± 5.4 kg, UI: 76.2 ± 4.0 kg; *p* = 0.94).

**Table 1:** Demographics of cSCI participants. S, speech; PO, physical/occupational; R, recreational; Ps, psychological; V, vocational; D, dietary

Level	Age	Sex	ASIA	Complete?	Trauma?	Months since Injury	Rehabilitation	Surgeries
C4	69	F	B	I	Y	99	S, PO, R, Ps, D	Spinal fusion
C4	28	M	D	I	Y	5	PO, R	Spinal fusion
C2-C6	21	F	–	I	N	20	PO, R, Ps	None
C5	21	M	B	I	Y	43	S, PO, R	Spinal fusion
C5-C6	34	M	–	I	Y	65	PO, R, Ps	Spinal fusion
C5-C6	41	M	B	I	Y	31	S, PO, R	Spinal fusion, suprapubic catheter
C6	22	M	B	I	Y	45	S, PO, R, Ps, V, D	Spinal fusion
C6	22	M	C	I	Y	57	PO	Spinal fusion
C6-C7	25	M	C	I	Y	40	S, PO, R	Spinal fusion
C7	41	F	–	I	Y	246	PO, R	Bilateral tendon transfer, autologous stem cell transplant
C7	45	F	–	I	Y	118	S, PO, Ps, V	Spinal fusion
C7	26	M	A	C	Y	54	PO, R Ps	Spinal fusion
C7	23	M	C	I	Y	84	PO	None

### Measurement validity

All force and ROM measurements and the statistical comparisons between the two participant groups are summarized in Table 2.

**Table 2:** Novel system measurement results by participant group (*N* = 13). CV, coefficient of variation; ^†^Values based on *n* = 12

		Peak			CV		
		UI	cSCI		UI	cSCI	
Measure	Task	Mean	Mean	p-val	Mean	Mean	p-val
	(units)	(SD)	(SD)		(SD)	(SD)	
Force	Finger Flexion	78.3	2.94	< 0.001	6.8	40.1	< 0.001
	(N)^†^	(22.7)	(2.49)		(2.3)	(25.3)	
	Finger Extension	17.3	1.23	< 0.001	12.7	71.1	< 0.001
	(N)^†^	(5.42)	(1.99)		(5.2)	(34.6)	
	Wrist Flexion	5.41	0.89	< 0.001	14.7	27.4	0.009
	(Nm)	(2.46)	(0.75)		(4.0)	(18.2)	
	Wrist Extension	3.24	1.27	< 0.001	12.4	25.7	0.002
	(Nm)	(1.29)	(0.98)		(4.6)	(12.8)	
	Handle Pronation	6.36	1.76	< 0.001	8.3	31.4	0.015
	(Nm)	(2.37)	(1.36)		(2.9)	(33.2)	
	Handle Supination	4.58	1.10	< 0.001	8.0	21.0	0.013
	(Nm)	(1.71)	(0.67)		(3.5)	(16.5)	
	Doorknob Pronation	3.63	0.30	< 0.001	10.3	60.7	< 0.001
	(Nm)	(1.14)	(0.28)		(3.6)	(44.9)	
	Doorknob Supination	3.51	0.38	< 0.001	11.6	28.6	< 0.001
	(Nm)	(1.40)	(0.32)		(5.9)	(13.3)	
Range of Motion	Wrist Flexion	81.3	56.5	0.027	4.0	8.4	0.11
	(°)	(5.56)	(26.7)		(2.7)	(7.8)	
	Wrist Extension (°)	71.9 (8.36)	48.5 (20.9)	0.002	2.7 (1.3)	7.6 (7.1)	0.026
	Handle Pronation	104.1	95.9	0.92	4.5	6.5	0.87
	(°)	(12.6)	(36.9)		(2.4)	(4.4)	
	Handle Supination	74.0	56.8	0.051	5.3	4.3	0.31
	(°)	(14.5)	(24.2)		(1.6)	(2.4)	
	Doorknob Pronation	107.1	94.7	0.72	4.4	21.7	0.12
	(°)	(20.9)	(41.5)		(2.4)	(36.7)	
	Doorknob Supination	72.1	56.7	0.25	5.4	36.8	0.13
	(°)	(19.8)	(35.1)		(2.4)	(69.7)	

Finger strength is essential for prehension, and it is the most commonly and severely impaired component of upper limb function after cSCI [9, 22–24]. As expected, participants with cSCI delivered significantly less flexion and extension force than uninjured participants (Fig. 2c) [25, 26]. Finger flexion force in cSCI participants was well-correlated with both grip and lateral pinch dynamometry, demonstrating concurrent validity (Flexion force v. grip strength, Pearson’s correlation, r^2^ = 0.73, *p* = 4 × 10^− 4^; Flexion force v. pinch strength, r^2^ = 0.66, *p* = 0.001). These results indicate that the finger isometric force device provides accurate measurements of pinch grip forces in individuals with cSCI.

**Fig. 3:**
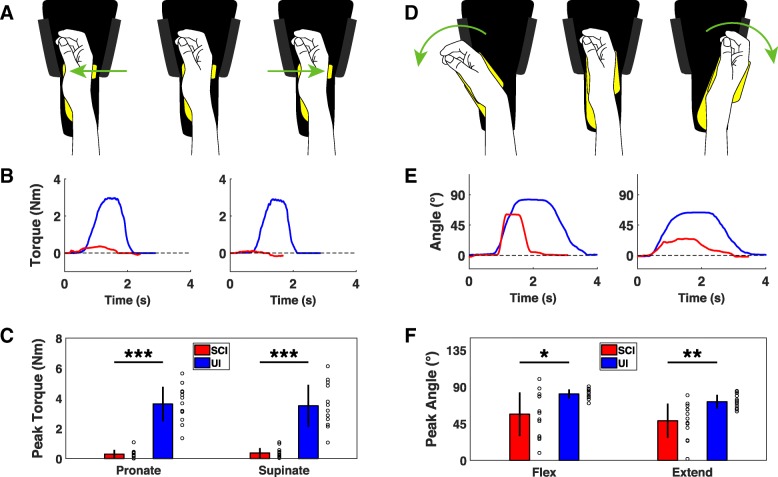
Devices for assessing wrist flexion and extension force and range of motion. **a** Diagram of isometric wrist force module. Red arrows indicate force direction. **b** Example of single wrist flexion and extension trials from uninjured and cSCI participants. **c** cSCI participants produce significantly lower wrist flexion and extension forces compared to uninjured controls. **d** Diagram of the isotonic wrist flexion and extension ROM device showing direction of movement. **e** Example of single flexion and extension ROM trials performed by uninjured and cSCI participants. **f** Wrist flexion and extension ROM is significantly reduced in cSCI participants compared to uninjured participants. Individual data is depicted with open circles. Error bars indicate SD. Significant differences were determined by Wilcoxon rank sum tests and are noted as *p < 0.05, **p < 0.01, ***p < 0.001

Wrist movement is also an essential element of prehension, thus we designed two devices to quantify torque and ROM of wrist flexion and extension [8, 27, 28]. cSCI participants produced significantly less isometric flexion and extension force than control participants, consistent with previous studies (Fig. 3c) [8, 17, 18, 29]. Similarly, both wrist flexion and extension ROM were significantly impaired compared to control participants (Fig. 3f) [30]. These results demonstrate that these two devices can reveal deficits in wrist flexion and extension function in individuals with cSCI.

**Fig. 4:**
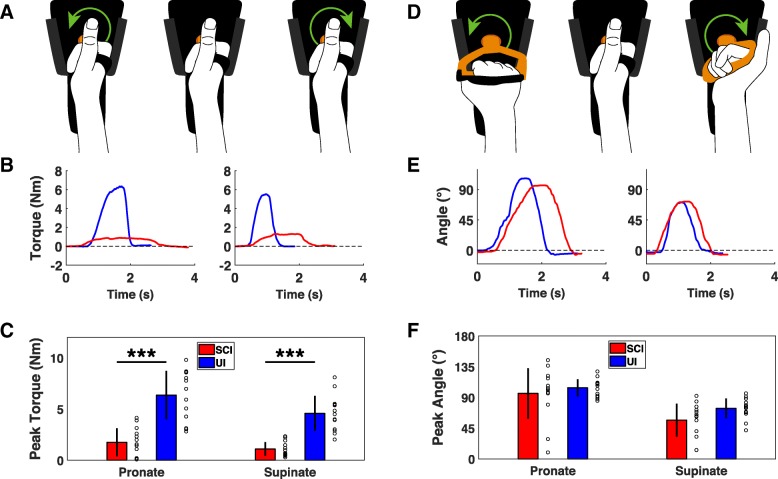
Assessment of wrist rotation function with the handle device. **a** Diagram of isometric wrist rotation module with the D-grip handle manipulandum. Green arrows indicate force direction. **b** Example of single isometric wrist pronation and supination trials from uninjured and cSCI participants. **c** Wrist pronation and supination force produced using the handle is significantly reduce in cSCI participants compared to uninjured controls. **d** Diagram of the isotonic wrist pronation and supination ROM device with the handle manipulandum illustrating direction of movement. **e** Example of single flexion and extension ROM trials performed with the handle by uninjured and cSCI participants. **f** cSCI participants exhibit comparable wrist pronation ROM and a small, but significant, reduction in wrist supination ROM. Individual data is depicted with open circles. Error bars indicate SD. Significant differences were determined by Wilcoxon rank sum tests and are noted as ***p < 0.001

**Fig. 5:**
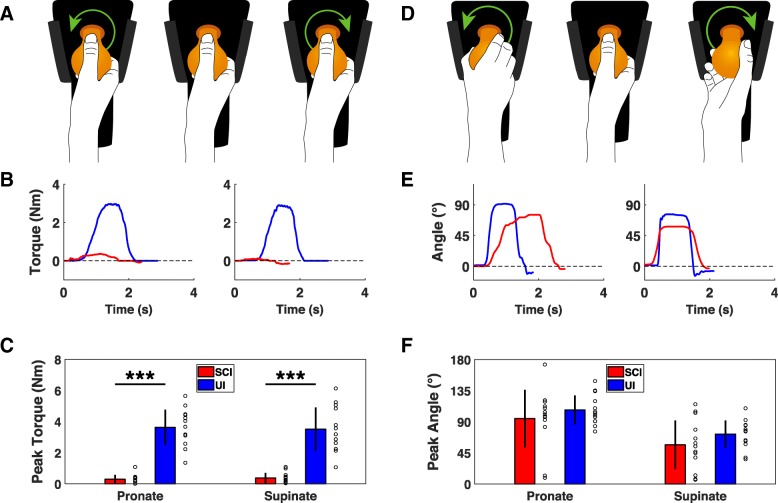
Assessment of wrist rotation function with the doorknob device. **a** Diagram of isometric wrist rotation module with the doorknob manipulandum. Green arrows indicate force direction. **b** Example of single isometric wrist pronation and supination trials from uninjured and cSCI participants collected using the doorknob manipulandum. **c** cSCI participants produce significantly less wrist pronation and supination force compared to uninjured controls. **d** Diagram of the isotonic wrist pronation and supination ROM device with the doorknob manipulandum illustrating direction of movement. **e** Example of single flexion and extension ROM trials performed with the doorknob by uninjured and cSCI participants. **f** Using the doorknob manipulandum, wrist pronation and supination ROM is not significantly impaired in cSCI participants compared to uninjured participants. Individual data is depicted with open circles. Error bars indicate SD. Significant differences were determined by Wilcoxon rank sum tests and are noted as ***p < 0.001

Lastly, we designed two devices to measure force and ROM of wrist pronation and supination [31, 32]. We assessed these metrics using two distinct manipulanda: an easy to grasp D-grip handle and a more difficult to grasp doorknob. cSCI participants produced significantly less handle pronation and supination force than control participants (Fig. 4c). Consistent with a greater difficulty in grasping the spherical manipulandum, the forces generated using the doorknob manipulandum were significantly lower than those produced with the handle (Fig. 5c; D-grip handle v. doorknob; Paired t-test; cSCI pronation: *p* = 0.001; cSCI supination: *p* < 0.001). Wrist pronation and supination ROM were less affected by SCI than force production. No differences were observed in pronation or supination ROM with the handle in cSCI patients compared to control participants (Fig. 4c). Similarly, no significant differences were observed between groups for pronation or supination with the doorknob (Fig. 5c). Altogether, these results suggest that isometric force measures of wrist rotation reveals deficits associated with cSCI while ROM is less affected.

**Fig. 6:**
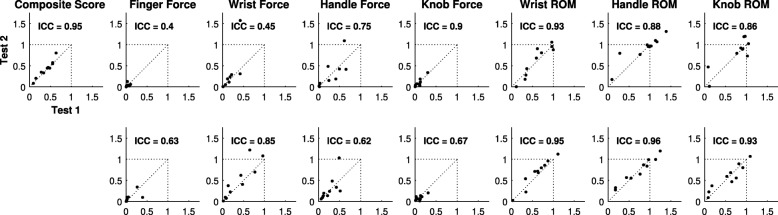
Test-retest reliability**.** Each panel depicts the correlation of each measure for the first and second test sessions. Individual task measurements from both test sessions are normalized to the mean value for the corresponding metric in uninjured subjects from the first test session. The composite score is the average of the normalized scores from all 14 individual metrics. The bottom row of panels represents extension or supination tasks. In each panel, individual cSCI participant data is depicted as black circles. ICC: intraclass correlation

**Fig. 7:**
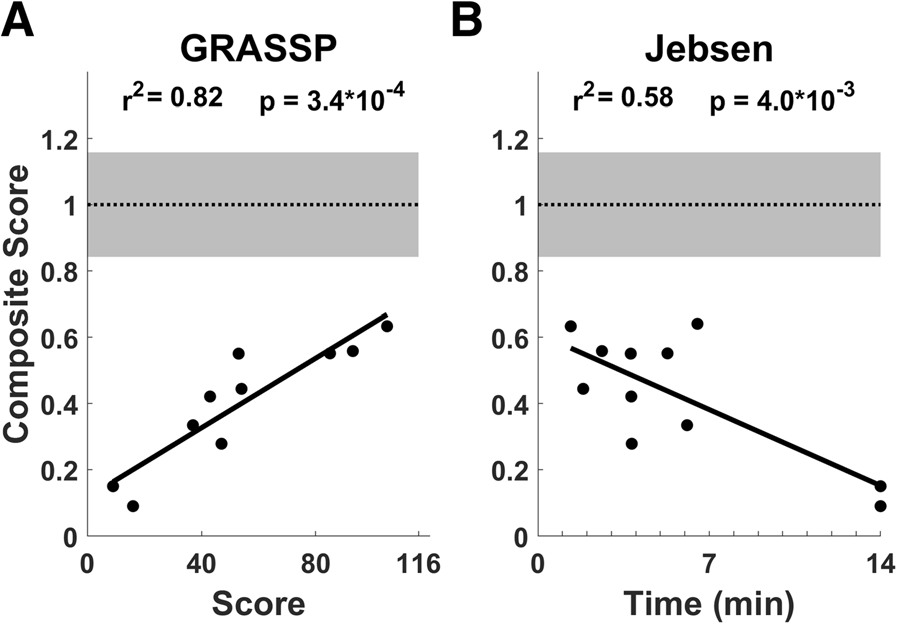
Motor function measured using the devices is correlated with standard assessments. Normalized composite score integrating performance on all devices is highly correlated with total GRASSP score (**a**) and moderately correlated with score on the Jebsen Hand Function Test (**b**). The grey areas indicate SD of the control group’s composite scores

### Measurement variance and minimal detectable difference

Coefficient of variation (CV) can be used to standardize measurement reliability across individuals. We calculated a CV based on the 10 trials acquired for each direction of movement on each device during the first test session. The average individual CV for all measurements and the statistical comparisons between the two participant groups are summarized in Table 2. Participants with cSCI demonstrated greater average CVs for all of the force measurements compared to uninjured participants. Alternatively, the average CVs for the majority of the ROM measurements were not significantly different between groups. These results demonstrate that participants with cSCI had greater trial-to-trial variability relative to mean performance on measures of force generation.

Calculating a detection limit is necessary for understanding a measurement’s sensitivity to real changes in performance. The minimal detectable difference (MDD) is used to determine the detection limits of an ordinal scale, which produces a single value per test session. Because the devices were able to record performance on multiple trials, we also characterized measurement variability across trials. For each of the metrics, we computed two values to determine the magnitude of change detectable outside of measurement error with 95% confidence: (1) the MDD, based on the variance of change in individuals’ mean performance between test sessions, and (2) the average SD of all 20 trials obtained from both test sessions (SD_20_). These test-retest reproducibility values for the cSCI group are provided in Table 3. The MDD and SD_20_ values of normalized measurements were highly correlated across all tasks (Pearson’s correlation, r^2^ = 0.77, *p* = 4.1 × 10^− 5^). Additionally, the SD values were consistently lower than the corresponding MDDs, indicating that evaluating more trials provides greater sensitivity to real changes in performance.

**Table 3:** Test-retest reproducibility results of the novel metrics for cSCI participants (*N* = 10). MDD, minimally detectable difference; SD_20_, standard deviation of 20 trials; ICC, intraclass correlation coefficient; ^†^Values based on *n* = 9

Measure	Task (units)	Change (SD)	p-val	MDD	1.96*SD_20_	ICC
	Composite Score	0.03 (0.06)	0.72	0.107	–	0.95
Force	Finger Flexion (N)^†^	0.21 (3.42)	0.88	5.99	2.52	0.40
	Finger Extension (N)^†^	−0.08 (1.99)	0.94	3.48	1.29	0.63
	Wrist Flexion (Nm)	0.65 (1.99)	0.45	3.70	0.96	0.45
	Wrist Extension (Nm)	0.34 (0.63)	0.55	1.17	0.68	0.85
	Handle Pronation (Nm)	0.13 (1.37)	0.88	2.55	1.10	0.75
	Handle Supination (Nm)	0.16 (0.16)	0.75	1.79	0.76	0.62
	Doorknob Pronation (Nm)	0.005 (0.16)	0.97	0.30	0.28	0.90
	Doorknob Supination (Nm)	−0.05 (0.22)	0.65	0.41	0.24	0.67
Range of Motion	Wrist Flexion (°)	1.52 (10.8)	0.90	20.1	10.8	0.93
	Wrist Extension (°)	4.46 (6.4)	0.67	11.9	8.3	0.95
	Handle Pronation (°)	3.49 (18.7)	0.84	34.7	15.5	0.88
	Handle Supination (°)	0.07 (7.6)	0.99	14.0	7.4	0.96
	Doorknob Pronation (°)	5.29 (20.8)	0.76	38.7	22.1	0.86
	Doorknob Supination (°)	0.03 (9.4)	0.99	17.5	14.9	0.93

### Test-retest reliability

To evaluate whether the devices provided reliable longitudinal assessment, participants were retested on the same assessments approximately 4 months later (*n* = 10). We generated a composite score to normalize and combine performance across all devices. Intraclass correlation coefficients (ICC) for each task and the composite score are provided in Table 3. The composite score and ROM tasks demonstrated excellent reliability (ICC > 0.8). As expected, performance on individual devices was comparatively less reliable than the composite score. Two of the force measurements for the cSCI participants were not reliable (ICC < 0.6), possibly due to the smaller measurement spread and higher CVs (Fig. 6). Overall, these findings indicate that the devices provide reliable, stable assessment over time to facilitate longitudinal testing.

### Concurrent validity

To benchmark these devices versus field-standard metrics and evaluate concurrent validity, we compared measurements from each device and the composite score to GRASSP and JHFT scores in cSCI participants. The two measurements acquired with each of the 7 devices were combined to simplify comparisons. One composite score was not included due to an incomplete measurement set (see Practical Utility section below). The composite score was highly correlated with total GRASSP scores (Device composite score v. GRASSP score, Pearson’s correlation, r^2^ = 0.82, *p* = 3.4 × 10^− 4^ Fig. 7). Additionally, the composite score was moderately correlated with JHFT score (Device composite score v. JHFT total score, Pearson’s correlation, r^2^ = 0.58, *p* = 0.004 Fig. 7). Individual correlations for each device can be found in the Additional file 1: Tables S1 and S2 and Additional file 2: Figure S1. These results indicate that measurements collected with the devices are well-correlated with standard metrics used in cSCI studies.

### Practical utility of the measurement devices in cSCI participants

All cSCI participants were able to use most of the devices with minimal difficulty. The only instance in which a participant was unable to interact with the devices was a participant with a C7 injury that could not use the finger force device due to severe proximal interphalangeal contractures that prohibited comfortable extension. One participant with a C5 injury was not able to complete the wrist flexion and doorknob ROM device assessments without assistance returning to the neutral position. Each force device required about 3–4 min to complete 20 trials including a one minute rest period, while each ROM assessment required approximately one minute. These results suggest that the system is practical for quickly and easily quantifying arm function in the context of chronic cSCI.

## Discussion

Here we provide a characterization of a novel suite of automated devices to measure hand and wrist motor function after cSCI. The system consists of seven distinct tasks that quantify various aspects of isometric force and single joint ROM. We compared measurements made with each of the devices in cSCI and uninjured participants, determined detection limits of each metric for participants with cSCI, evaluated reliability of measures over time, and correlated the metrics with established assessments of quadriplegic upper limb function.

Deficits in strength and range of motion of the arm impair prehension after cSCI [8, 9, 22, 33]. Accurate and sensitive measurement of functional impairment is critical for determining the efficacy of treatments focused on restoring motor function. The most common methods of arm assessment after cSCI include subjective categorization of isometric force and free range of motion, like in the ASIA and GRASSP exams [5]. These assessments use ordinal scoring with scales designed to balance measurement sensitivity and reproducibility and consequently demonstrate excellent inter-rater reliability [7, 34]. Sensor technology is capable of measuring motor function with greater precision, both because of a higher resolution scale and the capacity for a larger number of repeated trials within the same test session, and so numerous technologies have been developed to provide objective measurement of prehension [10]. Confining measurements to a single degree of freedom of movement limits sources of error, helping to reduce variability and improve sensitivity. Following this rationale, we restricted our measurements to well-controlled movements that together require the majority of the muscles in the hand and forearm.

Overall, the devices provided a robust characterization of impairments after cSCI. As expected, when compared to uninjured controls, cSCI participants were the most severely impaired on tasks that required finger strength, specifically the finger and doorknob isometric force devices [9]. The force metric that was least impaired compared to controls was wrist extension [35]. The majority of cSCI participants demonstrated ROM that was comparable to uninjured controls, indicating the isometric tasks provide a more robust assessment of motor deficits. Altogether, these results demonstrate that the devices described in this study are capable of providing accurate measurements across many distinct and essential wrist and finger functions commonly impaired after cSCI.

An essential feature of any assessment is its ability to detect significant changes outside of measurement error. The MDD is necessary to determine an assessment scale’s capacity to detect real changes when only a single sample is available per test session, as is the case for the GRASSP and JHFT assessments [21]. In contrast, the devices provided continuous data and enabled rapid collection of repeated trials within the same test session. To characterize the capacity of the devices to detect real changes in performance, we compared two distinct measures of within-subject variability: (1) the MDD, which is based on the variance of changes in individuals’ mean performance between test sessions, and (2) the average variance of all 20 trials acquired during both test sessions. For participants with cSCI, we found the 95% confidence detection limit based on variance was highly correlated with and reliably smaller than the MDD, consistent with the notion that sampling more trials increases the probability of identifying significant changes in performance. Furthermore, the acquisition of multiple trials per test session allows within-subject comparison, which increases statistical power.

Consistent, stable assessment is critical for longitudinal studies that rely on evaluating recovery over time. Overall, we find that the devices provide good test-retest reliability. All of the ROM measures, as well as a composite score that took into account performance on all devices, demonstrated excellent reliability. The force measures were less reliable, likely due to the relatively smaller measurement spread and larger CVs. These results suggest that the devices developed here may be useful for long-term studies aimed at improving hand and wrist function after cSCI.

Previous cSCI studies suggest that isolated components of upper limb strength can significantly contribute to task performance [8, 9, 36]. The metrics of hand and wrist function collected with the devices correlate well with gold-standard assessments used in SCI rehabilitation studies. The composite score was very highly correlated with the GRASSP score for the right arm. Of the four GRASSP subscores, quantitative prehension was best correlated with the composite score, demonstrating a strong relationship between execution of basic motor functions and functional task performance (Additional file 1: Table S2). Additionally, the composite score was well-correlated with the JHFT test. However, the correlation was slightly weaker than that observed for GRASSP scores, primarily driven by a more bimodal distribution of JHFT scores compared to a more even distribution of GRASSP and composite device scores. The strong correlations between the composite score and both gold-standard assessments suggests that relatively limited, simple assessment with the devices can be used to accurately gauge hand and wrist function after cSCI.

The assessment devices described in this study were developed to provide a number of advantages over existing tests of function for rehabilitation studies. One key advantage is the simple measurement of continuous data in physical units, including angle, torque, and linear force. This allows direct comparison of effect size and magnitude of changes in performance, a benefit over assessments that rely on ordinal scores. The use of continuous physical values also largely mitigates any variance in tests that rely on a subjective scoring system, which would facilitate comparison of data across multiple sites in a trial. Moreover, unlike ordinal assessments, there is no performance ceiling or necessary stratification, which may make the devices useful for measuring hand and wrist function in other populations with varying degrees of impairment, such as stroke patients.

Measurement with the devices is simple and relatively rapid. Data collection with the entire suite of devices took approximately twenty minutes to collect ten repeats of each movement in each direction, a total of 140 trials. This testing duration is comparable to or slightly shorter than that typically required for GRASSP and JHFT, which respectively require approximately thirty or fifteen minutes to complete for each hand. While the purpose of this study was to collect comprehensive data on all devices, selecting a subset of devices based on residual upper limb function and reducing the number of repeats to match the desired statistical power could potentially speed data collection further. Finally, the system is compact in size and constructed from low-cost components. This raises the potential for the devices to be packaged for home use. The simple data stream collected with the devices could also be easily implemented into a video game architecture to increase engagement and promote user compliance [37–39].

While the devices provide a number of advantages for quantitative rehabilitation studies, one disadvantage is the absence of direct measurement of sensory function, which would need to be supplemented with an additional assessment. Another disadvantage is the restriction to movement about a single joint. Given the complexity and fine motor control of prehension, this restriction to a small number of single joint motions fails to capture the full range of hand and wrist dysfunction. However, constraining the complexity of movement simplifies and improves measurement capabilities [9, 17]. Future studies are required to directly correlate device measures with functional outcomes, including ability to perform activities of daily living.

## Conclusion

In this study, we characterize a set of tools to quantify hand and wrist function after spinal cord injury. We report that these devices provide accurate, stable measurement of isometric and isotonic function and are well-correlated with gold-standard assessments. These results indicate that these tools represent a reliable system for longitudinal assessment of upper extremity function after cSCI and may provide a framework to assess the efficacy of strategies aimed at improving recovery of upper limb function.

## Additional files

Additional file 1: **Table S1.** Individual devices and standard assessments are correlated. ^†^Given in units of metric/assessment. **Table S2.** Composite performance and standard assessments are well-correlated. ^†^Given in units of composite score/assessment. (DOCX 19 kb)

Additional file 2: **Figure S1.** Novel metrics correlate with GRASSP. Each of the 7 normalized metrics are positively correlated with GRASSP score. The novel metrics demonstrate a diverse range of motor function impairments after cSCI. (PDF 11 kb)

## Abbreviations

cSCI: cervical spinal cord injury
CV: Coefficient of variation
GRASSP: Graded Redefined Assessment of Strength, Sensitivity and Prehension
ICC: Intraclass correlation coefficient
JHFT: Jebsen Hand Function Test
PCB: Printed circuit board
ROM: Range of motion
UI: Uninjured
