# Correction to: A suite of automated tools to quantify hand and wrist motor function after cervical spinal cord injury

**DOI:** 10.1186/s12984-019-0627-4

**Published:** 2020-01-07

**Authors:** Katelyn M. Grasse, Seth A. Hays, Kimiya C. Rahebi, Victoria S. Warren, Elizabeth A. Garcia, Jane G. Wigginton, Michael P. Kilgard, Robert L. Rennaker

**Affiliations:** 1 The University of Texas at Dallas, Texas Biomedical Device Center800 West Campbell Road, Richardson, TX 75080-3021 USA; 2 The University of Texas at Dallas, Erik Jonsson School of Engineering and Computer Science, 800 West Campbell Road, Richardson, TX 75080-3021 USA; 3 The University of Texas at Dallas, School of Behavioral Brain Sciences, 800 West Campbell Road, Richardson, TX 75080-3021 USA

**Correction to: J NeuroEng Rehabil (2019) 16:48**

**https://doi.org/10.1186/s12984-019-0518-8**

The original article [1] contains several errors which the authors would like to rectify:

1. Figs 3B & 3C contain duplicate data from Fig. 5. The correct version of Fig. 3 can be viewed ahead.
2. The **Authors’ contributions** section contains a minor typo and should instead read as the following:
  “KMG, SAH, JW, MPK and RLR contributed to experimental design. KMG and SAH wrote the manuscript. KMG and RLR provided engineering. KMG, KR, VW and EG conducted data collection. SAH, MPK, and RLR provided funding. All authors read and approved the final manuscript.”
3. Tables 2 & 3 contain minor formatting errors. The correct presentation of both tables can be viewed ahead.

**Fig. 3:**
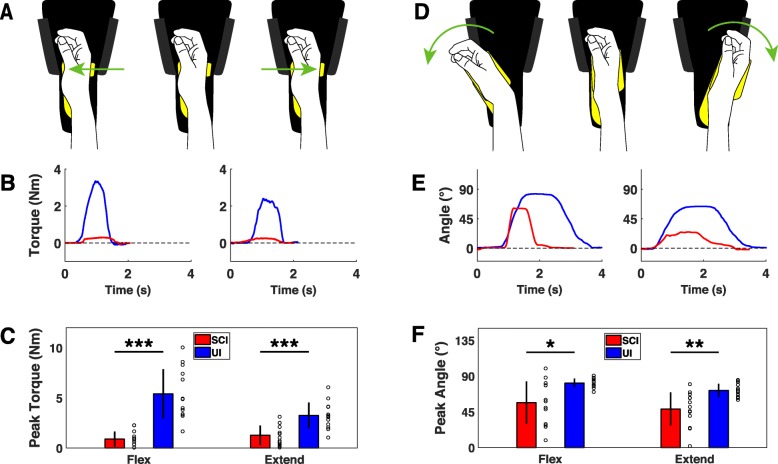
Devices for assessing wrist flexion and extension force and range of motion. **a** Diagram of isometric wrist force module. Red arrows indicate force direction. **b** Example of single wrist flexion and extension trials from uninjured and cSCI participants. **c** cSCI participants produce significantly lower wrist flexion and extension forces compared to uninjured controls. **d** Diagram of the isotonic wrist flexion and extension ROM device showing direction of movement. **e** Example of single flexion and extension ROM trials performed by uninjured and cSCI participants. **f** Wrist flexion and extension ROM is significantly reduced in cSCI participants compared to uninjured participants. Individual data is depicted with open circles. Error bars indicate SD. Significant differences were determined by Wilcoxon rank sum tests and are noted as *p < 0.05, **p < 0.01, ***p < 0.001

**Table 2:** Novel system measurement results by participant group (N = 13). CV, coefficient of variation; †Values based on *n* = 12

		Peak			CV		
		UI	cSCI		UI	cSCI	
Measure	Task (units)	Mean (SD)	Mean (SD)	*p*-val	Mean (SD)	Mean (SD)	*p*-val
Force	Finger Flexion (N)^†^	78.3 (22.7)	2.94 (2.49)	< 0.001	6.8 (2.3)	40.1 (25.3)	< 0.001
	Finger Extension (N)^†^	17.3 (5.42)	1.23 (1.99)	< 0.001	12.7 (5.2)	71.1 (34.6)	< 0.001
	Wrist Flexion (Nm)	5.41 (2.46)	0.89 (0.75)	< 0.001	14.7 (4.0)	27.4 (18.2)	0.009
	Wrist Extension (Nm)	3.24 (1.29)	1.27 (0.98)	< 0.001	12.4 (4.6)	25.7 (12.8)	0.002
	Handle Pronation (Nm)	6.36 (2.37)	1.76 (1.36)	< 0.001	8.3 (2.9)	31.4 (33.2)	0.015
	Handle Supination (Nm)	4.58 (1.71)	1.10 (0.67)	< 0.001	8.0 (3.5)	21.0 (16.5)	0.013
	Doorknob Pronation (Nm)	3.63 (1.14)	0.30 (0.28)	< 0.001	10.3 (3.6)	60.7 (44.9)	< 0.001
	Doorknob Supination (Nm)	3.51 (1.40)	0.38 (0.32)	< 0.001	11.6 (5.9)	28.6 (13.3)	< 0.001
Range of Motion	Wrist Flexion (°)	81.3 (5.56)	56.5 (26.7)	0.027	4.0 (2.7)	8.4 (7.8)	0.11
	Wrist Extension (°)	71.9 (8.36)	48.5 (20.9)	0.002	2.7 (1.3)	7.6 (7.1)	0.026
	Handle Pronation (°)	104.1 (12.6)	95.9 (36.9)	0.92	4.5 (2.4)	6.5 (4.4)	0.87
	Handle Supination (°)	74.0 (14.5)	56.8 (24.2)	0.051	5.3 (1.6)	4.3 (2.4)	0.31
	Doorknob Pronation (°)	107.1 (20.9)	94.7 (41.5)	0.72	4.4 (2.4)	21.7 (36.7)	0.12
	Doorknob Supination (°)	72.1 (19.8)	56.7 (35.1)	0.25	5.4 (2.4)	36.8 (69.7)	0.13

**Table 3:** Test-retest reproducibility results of the novel metrics for cSCI participants (*N* = 10). MDD, minimally detectable difference; SD_20_, standard deviation of 20 trials; ICC, intraclass correlation coefficient; ^†^Values based on *n* = 9

Measure	Task (units)	Change (SD)	*p*-val	MDD	1.96*SD_20_	ICC
	Composite Score	0.03 (0.06)	0.72	0.107	–	0.95
Force	Finger Flexion (N)^†^	0.21 (3.42)	0.88	5.99	2.52	0.40
	Finger Extension (N)^†^	−0.08 (1.99)	0.94	3.48	1.29	0.63
	Wrist Flexion (Nm)	0.65 (1.99)	0.45	3.70	0.96	0.45
	Wrist Extension (Nm)	0.34 (0.63)	0.55	1.17	0.68	0.85
	Handle Pronation (Nm)	0.13 (1.37)	0.88	2.55	1.10	0.75
	Handle Supination (Nm)	0.16 (0.16)	0.75	1.79	0.76	0.62
	Doorknob Pronation (Nm)	0.005 (0.16)	0.97	0.30	0.28	0.90
	Doorknob Supination (Nm)	−0.05 (0.22)	0.65	0.41	0.24	0.67
Range of Motion	Wrist Flexion (°)	1.52 (10.8)	0.90	20.1	10.8	0.93
	Wrist Extension (°)	4.46 (6.4)	0.67	11.9	8.3	0.95
	Handle Pronation (°)	3.49 (18.7)	0.84	34.7	15.5	0.88
	Handle Supination (°)	0.07 (7.6)	0.99	14.0	7.4	0.96
	Doorknob Pronation (°)	5.29 (20.8)	0.76	38.7	22.1	0.86
	Doorknob Supination (°)	0.03 (9.4)	0.99	17.5	14.9	0.93
