# Supplementary material for: A suite of automated tools to quantify hand and wrist motor function after cervical spinal cord injury
Source: J Neuroeng Rehabil. 2019 Apr 11;16:48. doi: 10.1186/s12984-019-0518-8 (PMC6458684; doi:10.1186/s12984-019-0518-8)
Supplement: Supplementary file 1 — Table S1. Individual devices and standard assessments are correlated. †Given in units of metric/assessment. Table S2. Composite performance and standard assessments are well-correlated. †Given in units of composite score/assessment. (DOCX 19 kb) [file 12984_2019_518_MOESM1_ESM.docx]

**Additional file 2**

|  | **GRASSP (pts)** | | | **Jebsen (min)** | | |
| --- | --- | --- | --- | --- | --- | --- |
| **Metric (units)** | **r^2^** | **p-val** | **slope**^†^ | **r^2^** | **p-val** | **slope**^†^ |
| **Pinch Force (N)** | 0.77 | <0.001 | 0.066 | 0.24 | 0.13 | -0.25 |
| **Wrist Force (Nm)** | 0.33 | 0.063 | 0.015 | 0.29 | 0.07 | -0.11 |
| **Handle Force (Nm)** | 0.48 | 0.018 | 0.024 | 0.37 | 0.036 | -0.15 |
| **Knob Force (Nm)** | 0.78 | <0.001 | 0.008 | 0.41 | 0.025 | -0.045 |
| **Wrist ROM (º)** | 0.83 | <0.001 | 0.64 | 0.49 | 0.011 | -3.8 |
| **Handle ROM (º)** | 0.41 | 0.035 | 0.46 | 0.51 | 0.009 | -3.9 |
| **Knob ROM (º)** | 0.66 | 0.002 | 0.82 | 0.47 | 0.014 | -5.9 |

**Table S1. Individual devices and standard assessments are correlated.** ^†^Given in units of metric/assessment.

| **Assessment (max score)** | **r^2^** | **p-val** | **slope**^†^ |
| --- | --- | --- | --- |
| **GRASSP Total Score (116)** | 0.82 | <0.001 | 0.0065 |
| **GRASSP Strength (50)** | 0.53 | 0.017 | 0.022 |
| **GRASSP Sensation (24)** | 0.79 | <0.001 | 0.023 |
| **GRASSP Qual. Prehension (12)** | 0.61 | 0.008 | 0.058 |
| **GRASSP Quant. Prehension (30)** | 0.84 | <0.001 | 0.02 |
| **Jebsen Time (14)** | 0.58 | 0.004 | -0.056 |

**Table S2. Composite performance and standard assessments are well-correlated.** ^†^Given in units of composite score/assessment.
