# Supplementary material for: A suite of automated tools to quantify hand and wrist motor function after cervical spinal cord injury
Source: J Neuroeng Rehabil. 2019 Apr 11;16:48. doi: 10.1186/s12984-019-0518-8 (PMC6458684; doi:10.1186/s12984-019-0518-8)
Supplement: Supplementary file 2 — Figure S1. Novel metrics correlate with GRASSP. Each of the 7 normalized metrics are positively correlated with GRASSP score. The novel metrics demonstrate a diverse range of motor function impairments after cSCI. (PDF 11 kb) [file 12984_2019_518_MOESM2_ESM.pdf]

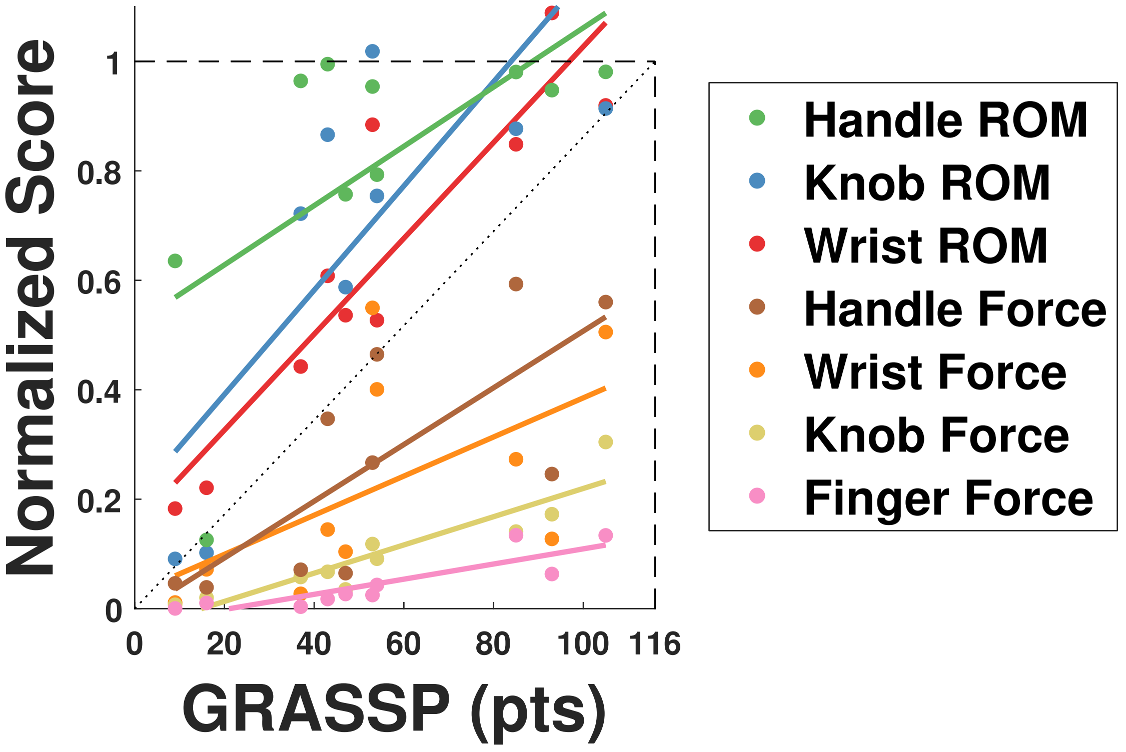

Normalized Score
0
0.2
0.4
0.6
0.8
1
0
20
40
60
80
100
116
GRASSP (pts)
Handle ROM
Knob ROM
Wrist ROM
Handle Force
Wrist Force
Knob Force
Finger Force
